# PAPSS2 Deficiency Causes Androgen Excess via Impaired DHEA Sulfation—In Vitro and in Vivo Studies in a Family Harboring Two Novel PAPSS2 Mutations

**DOI:** 10.1210/jc.2014-3556

**Published:** 2015-01-16

**Authors:** Wilma Oostdijk, Jan Idkowiak, Jonathan W. Mueller, Philip J. House, Angela E. Taylor, Michael W. O'Reilly, Beverly A. Hughes, Martine C. de Vries, Sarina G. Kant, Gijs W. E. Santen, Annemieke J. M. H. Verkerk, André G. Uitterlinden, Jan M. Wit, Monique Losekoot, Wiebke Arlt

**Affiliations:** Department of Pediatrics (W.O., M.C.d.V., J.M.W.), Leiden University Medical Center, 2300 RC Leiden, The Netherlands; Centre for Endocrinology, Diabetes, and Metabolism (J.I., J.W.M., P.J.H., A.E.T., M.W.O., B.A.H., W.A.), School of Clinical and Experimental Medicine, University of Birmingham, Birmingham B15 2TT, United Kingdom; Department of Clinical Genetics (S.G.K., G.W.E.S., M.L.), Leiden University Medical Center, 2300 RC Leiden, The Netherlands; and Department of Internal Medicine (A.J.M.H.V., A.G.U.), Erasmus Medical Center Rotterdam, 3000 CA Rotterdam, The Netherlands

## Abstract

**Context:**

PAPSS2 (PAPS synthase 2) provides the universal sulfate donor PAPS (3′-phospho-adenosine-5′-phosphosulfate) to all human sulfotransferases, including SULT2A1, responsible for sulfation of the crucial androgen precursor dehydroepiandrosterone (DHEA). Impaired DHEA sulfation is thought to increase the conversion of DHEA toward active androgens, a proposition supported by the previous report of a girl with inactivating PAPSS2 mutations who presented with low serum DHEA sulfate and androgen excess, clinically manifesting with premature pubarche and early-onset polycystic ovary syndrome.

**Patients and Methods:**

We investigated a family harboring two novel *PAPSS2* mutations, including two compound heterozygous brothers presenting with disproportionate short stature, low serum DHEA sulfate, but normal serum androgens. Patients and parents underwent a DHEA challenge test comprising frequent blood sampling and urine collection before and after 100 mg DHEA orally, with subsequent analysis of DHEA sulfation and androgen metabolism by mass spectrometry. The functional impact of the mutations was investigated in silico and in vitro.

**Results:**

We identified a novel *PAPSS2* frameshift mutation, c.1371del, p.W462Cfs*3, resulting in complete disruption, and a novel missense mutation, c.809G>A, p.G270D, causing partial disruption of DHEA sulfation. Both patients and their mother, who was heterozygous for p.W462Cfs*3, showed increased 5α-reductase activity at baseline and significantly increased production of active androgens after DHEA intake. The mother had a history of oligomenorrhea and chronic anovulation that required clomiphene for ovulation induction.

**Conclusions:**

We provide direct in vivo evidence for the significant functional impact of mutant PAPSS2 on DHEA sulfation and androgen activation. Heterozygosity for *PAPSS2* mutations can be associated with a phenotype resembling polycystic ovary syndrome.

Dehydroepiandrosterone (DHEA) can be converted to its inactive sulfate ester, DHEA sulfate (DHEAS), or toward active androgens via androstenedione and T to the most potent androgen, 5α-dihydrotestosterone (DHT). It was previously assumed that DHEA and DHEAS are continuously interconverted, with DHEAS serving as a circulating pool for reactivation to DHEA, and ultimately sex steroids. However, this concept was called into question by studies suggesting that DHEA sulfation by the enzyme DHEA sulfotransferase, SULT2A1, is the predominant reaction, and the conversion back to DHEA through the enzyme steroid sulfatase is only a rare occurrence (1, 2), except for distinct tissues with ample steroid sulfatase activity, such as placenta and cancers of prostate, breast, endometrium, and colon (3).

We previously described a female patient with clinical and biochemical evidence of androgen excess and concurrently very low serum DHEAS (4). She presented with premature pubarche at 6 years of age and then progressed to a clinically overt polycystic ovary syndrome (PCOS) phenotype, with acne, hirsutism, and eventually secondary amenorrhea at the age of 13 years. We hypothesized that impaired DHEA sulfation would explain the concurrent findings of low DHEAS and increased active androgens. Genetic analysis revealed compound heterozygous mutations in the *PAPSS2* gene encoding human PAPS synthase 2, which provides the sulfate donor PAPS (3′-phospho-adenosine-5′-phosphosulfate) to all human sulfotransferases including SULT2A1 (Figure 1A). Functional in vitro analysis of the mutant PAPSS2 proteins demonstrated significantly impaired DHEA sulfation (4).

**Figure 1.**
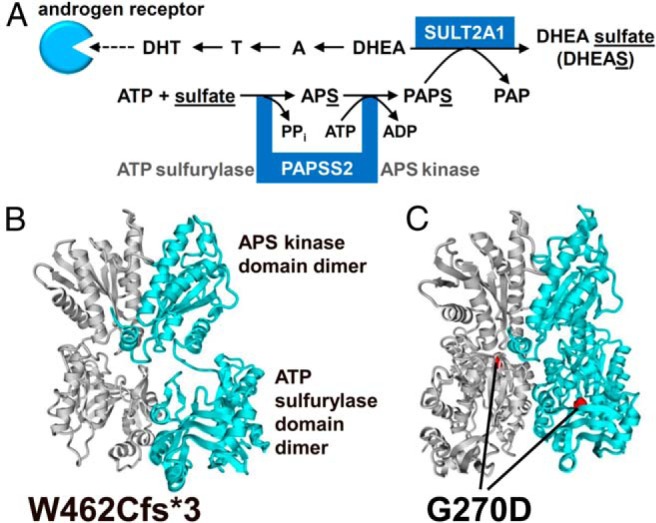
In silico analysis of the mutant PAPSS2 proteins. A, Either DHEA is converted via to T and DHT, activating the androgen receptor, or DHEA is sulfated by DHEA sulfotransferase (SULT2A1), which requires provision of the universal sulfate donor PAPS, generated by successive ATP sulfurylase and APS kinase activities of PAPSS2. A, androstenedione; APS, adenosine 5′-phosphosulfate; PAPS, 3′-phosphoadenosine 5′-phosphosulfate; PAP, 3′-phosphoadenosine 5′-phosphate; PPi, pyrophosphate. B and C, Homology model of human PAPSS2 based on the 1XNJ structure of PAPSS1 (12), with one high-affinity dimer represented in gray, the other in cyan, visualizing protein truncation by the frameshift mutation p.W462Cfs*3 (B) and the glycine residue affected by the missense mutation p.G270D (C).

Interestingly, homozygous *PAPSS2* mutations had already been described in 1998 in a consanguineous Pakistani family presenting with spondyloepimetaphyseal dysplasia (SEMD) (5, 6), a subgroup of the large and heterogeneous group of bone dysplasias (7), whereas no clinically overt bone phenotype was found in our female patient (4), with only mild radiological evidence of platyspondyly within the thoracic spine. The individuals in the Pakistani family, 11 men and five women, did not undergo endocrine investigations, and no access was granted to the women for clinical assessment. Three recent papers have described 24 additional individuals with PAPSS2 deficiency (8–10), all of them presenting with clinically overt bone dysplasia. However, serum androgens were measured in only five of them, unanimously revealing low DHEAS but normal circulating active androgens.

Here we have studied the biochemical and clinical consequences of PAPSS2 deficiency in a family with two brothers compound heterozygous for two novel *PAPSS2* mutations, who presented with clinically overt SEMD, low serum DHEAS, but normal serum androgens. We carried out an integrated in silico and in vitro mutation analysis as well as detailed in vivo studies of DHEA sulfation capacity and androgen synthesis in the patients and their heterozygous parents.

## Patients and Methods

### Patients

The first index case, P1, was born after an uneventful pregnancy at 37 weeks gestation (birth weight, 2580 g; −1.0 SD score [SDS]); birth length not recorded). Short stature with disproportionately short arms and legs was noticed during the first few months after birth. Psychomotor development was normal. At 4.6 years of age, he underwent detailed review by a clinical geneticist, documenting disproportionate growth and short stature with scoliosis, consistent with the phenotypic features of SEMD. His growth curve is shown in Supplemental Figure 1A. A skeletal survey showed scoliosis, platyspondyly with coronal clefts, horizontal acetabular roofs with small iliac wings, small femoral epiphyses, and slight metaphyseal changes, but normal hands (Supplemental Figure 2, A–D).

The patient reported normal age of pubarche (Tanner stage P2 at 12.5 y). At the age of 17.6 years, his height was 145.8 cm (−5.0 SDS), weight was 48.2 kg (body mass index [BMI], 0.7 SDS), head circumference was 56.8 cm (−0.1 SDS), arm span was 150.8 cm, sitting height was 78.5 cm, providing a sitting height/height ratio of 0.54 (+1.9 SDS), and his bone age was 15.5 years. Pubertal development was normal (Tanner stage A2P5G5; testicular volume, 25 mL bilaterally). Serum DHEAS was detectable but low (0.5 μmol/L; male reference range, 2.0–15.0 μmol/L); serum and T were within the reference range (Supplemental Table 1).

His brother, P2, who was 2.5 years younger, was found to have short extremities upon prenatal ultrasound. He was born at 38 weeks gestation (birth weight, 2860 g [−0.8 SDS]; length not recorded). Psychomotor development was normal. His growth curve is shown in Supplemental Figure 1B. Age of pubarche was normal (12–13 y). A skeletal survey showed skeletal abnormalities similar to his brother, P1 (Supplemental Figure 2, E–H). At the age of 15.1 years, his height was 143.3 cm (−4.1 SDS), weight was 45.3 kg (BMI, 1.3 SDS), span was 150.0 cm, sitting height was 73.7 cm (sitting height/height ratio, +0.3 SDS), and his bone age was 13.5 years. Pubertal development was normal (Tanner stage A1P4G4; testicular volume, 14 mL bilaterally). Serum DHEAS was low (0.44 μmol/L); circulating androgens were normal (Supplemental Table 1).

The nonconsanguineous parents had normal body proportions and height (father, 183 cm, −0.1 SDS; mother, 168 cm, −0.4 SDS). The 43-year-old mother reported a normal age of menarche (13 y), but irregular periods from the start. Due to anovulatory oligomenorrhea, all her pregnancies were only achieved after ovulation induction with clomiphene. She did not suffer from acne or hirsutism, and her ovaries did not appear polycystic upon imaging. At the age of 40 years, she developed psoriatic arthritis, and subsequently intolerance to several drugs was noted (for details, see Supplemental Data).

The older sister of the two index patients was of normal height (166 cm, −0.7 SDS) and weight (BMI, 23.2 kg/m^2^ at 19 years); however, she had failed to develop a spontaneous menarche and therefore had been initiated on oral contraceptives for cycle regulation at 13 years of age.

### Genetic analysis

Exome sequencing was undertaken after obtaining appropriate informed consent. Genomic DNA was isolated from peripheral blood using the AUTOPURE LS Instrument (Gentra Systems). Cytogenetic microarray analysis was performed using the CytoScan HD Array (Affymetrix) according to the manufacturer's protocol. Copy number was assessed using Chromosome Analysis Suite software (Affymetrix). Whole exome sequencing was performed on DNA fragmented into 200- to 400-bp fragments using Adaptive Focused Acoustics (Covaris Inc) shearing according to the manufacturer's instructions. The exome was captured by Nimblegen SeqCap EZ V2 kit (Roche Nimblegen, Inc) in combination with Illumina paired end library preparation and 2 × 100 bp sequencing with at least 70x mean coverage. Downstream analyses included demultiplexing (CASAVA software; Illumina Inc), sequence quality control, capture quality control, single nucleotide polymorphism calling, and indel (insertions and deletions) calling using different software applications as described by Santen (11). Identified mutations were confirmed by Sanger sequencing (primer sequences available on request).

### In silico modeling of the mutant PAPSS2 protein

A homology model of human PAPSS2 was built on the known crystal structure 1XNJ of PAPSS1 (12), as described previously (13), and the affected Gly270 residue was visualized using YASARA (14). A sequence alignment of 101 vertebrate and invertebrate sequences (15) was extended to plant, fungal, and yeast sequences of ATP sulfurylase. The alignment was created using Clustal W (16).

### In vitro functional analysis of the mutant PAPSS2 proteins

Expression vectors containing the human PAPSS2 coding sequence harboring the newly identified mutations were created by site-directed mutagenesis with *Dpn*I selection in the pcDNA6 and pEGFP-N1 vector backgrounds, as previously described (17).

To assess the impact of the mutant proteins on DHEA sulfation, an in-house clone of HEK293 cells with very low endogenous *PAPSS2* and *SULT2A1* expression was cotransfected with *PAPSS2* and *SULT2A1*; in addition to wild-type *PAPSS2* and the two novel *PAPSS2* mutants, we expressed the previously described p.T48R mutant (4) for comparison. Cells were incubated with 250 nm DHEA and 0.2 μCi ^3^H-DHEA for 2 hours at 37°C; all assays were performed in triplicate. Steroids were extracted as previously described (4) and analyzed on a LablogicAR2000 bioscanner. The conversion rate observed after expression of SULT2A1 alone was subtracted from those observed after coexpression with PAPSS2. For SULT2A1 detection by Western blotting, we used a polyclonal rabbit antibody ab38416 (Abcam), employing the horseradish peroxidase-linked β-actin monoclonal antibody ab20272 (Abcam) for confirmation of equal loading. PAPSS2 protein expression was tested using the anti-PAPSS2 monoclonal antibody ab56393 (Abcam) raised against amino acids 513–613 of PAPSS2, thus not suitable for detection of p.W462Cfs*3. Expression of p.W462Cfs*3 was confirmed at the mRNA level by real-time PCR using the Hs00989921_m1TaqMan probe for PAPSS2 (Life Technologies).

To test the impact of ubiquitination on the expression levels of the p.T48R and p.G270D mutants, these were separately expressed in HEK293 cells, followed by 6-hour incubation with 10 μm of the proteasome inhibitor MG-132 (Sigma) dissolved in dimethylsulfoxide or dimethylsulfoxide alone.

### DHEA challenge test

We employed our previously established in vivo physiology assessment tool, the DHEA challenge test (18, 19), to study in vivo DHEA sulfation and androgen synthesis in the two affected brothers and their heterozygous parents; the sister did not participate because she continued on an oral contraceptive, which precluded detailed analysis of androgen status. In brief, after obtaining written informed consent and with approval from the local ethics committee, all four individuals underwent blood sampling at baseline (9 am) and 30, 60, 90, 120, 180, and 240 minutes after the oral administration of 100 mg DHEA. All four participants collected two consecutive 24-hour urine samples, the first during the 24 hours preceding the DHEA challenge test, and the second starting at the time of DHEA administration.

### Serum and urine steroid measurements

Serum steroids were measured by liquid chromatography/tandem mass spectrometry employing a Waters Xevo mass spectrometer with Acquity uPLC system as described previously (20). In brief, serum steroid oxime analysis, which facilitates enhanced detection of DHEA by formation of oxime derivatives of the steroid oxo-groups (21), was employed for the measurement of DHEA, androstenedione, T, and DHT and carried out in positive mode, whereas measurement of serum DHEAS was performed in negative mode. DHEA, androstenedione, T, and DHT were extracted from serum via liquid-liquid extraction followed by derivatization into steroid oximes. Steroids were identified by matching retention times and two mass transitions in comparison to deuterated reference compounds.

Urinary steroid metabolite excretion analysis was carried out by quantitative gas chromatography/mass spectrometry in selected-ion-monitoring analysis mode, as described previously (22). In brief, free and conjugated urinary steroids were extracted by solid-phase extraction, and the conjugates were enzymatically hydrolyzed, followed by recovery of the hydrolyzed steroids by Sep-Pak (Waters) extraction. Specifically, we quantified 5α-reduced androsterone and 5β-reduced etiocholanolone, the two major metabolites of active androgens, which were used to assess net systemic 5α-reductase activity by calculating the ratio of androsterone/etiocholanolone (An/Et). We also measured urinary DHEA, which represents the sum of urinary DHEA and DHEAS excretion, which cannot be distinguished with this method because the hydrolysis step removes the sulfate group. However, in preceding experiments (data not shown) we found that >99% of urinary DHEA originates from DHEAS, ie, proportionate to their respective circulating serum concentrations in the nanomolar and micromolar range, respectively. Thus, for clarity purposes, we have labeled the sum of urinary DHEA and DHEAS in results and figures as urinary DHEAS.

### Statistical analysis

SPSS version 21 software (SPSS Inc) was used for data analysis. All data were expressed as mean ± SD unless otherwise stated. Independent samples *t* tests or Mann-Whitney tests were used as appropriate for comparison between two groups. Differences were considered statistically significant at *P* < .05.

## Results

### Identification of novel *PAPSS2* mutations

*FGFR3* and *COL2A1* mutations as distinct causes of the disproportionate short stature had been excluded by direct sequencing during childhood. When the affected brothers were 17 and 15 years old, whole exome sequencing was undertaken in all five family members. This identified compound heterozygosity for two novel *PAPSS2* mutations in both boys: a missense mutation (c.809G>A; p.Gly270Asp; p.G270D) and a frameshift mutation (c.1369del; p.W462Cfs*3). The mother was identified as a heterozygous carrier of p.W462Cfs*3, whereas the father and the sister were heterozygous for p.G270D.

### In silico analysis of the predicted mutant PAPSS2 proteins

Both novel mutations affect the ATP sulfurylase domain of the PAPSS2 protein. The p.W462Cfs*3 frameshift mutation will result in a severely truncated protein (Figure 1B). The p.G270D missense mutation affects a buried glycine residue within the ATP sulfurylase domain (Figure 1C), which is positioned in a short and bent β-strand of the PAPSS2 protein, 25Å apart from the bound adenosine 5′-phosphosulfate nucleotide in the 1XNJ structure of PAPSS1 (12) (http://www.rcsb.org/pdb/explore/explore.do?structureId=1xnj). This glycine is invariant in an alignment of 101 vertebrate and invertebrate PAPS synthases (15). Exchanging this highly conserved, small amino acid for a larger and negatively charged aspartic acid, as in p.G270D, is highly likely to impact on protein stability and function.

### In vitro functional analysis of the mutant PAPSS2 proteins

**Figure 2.**
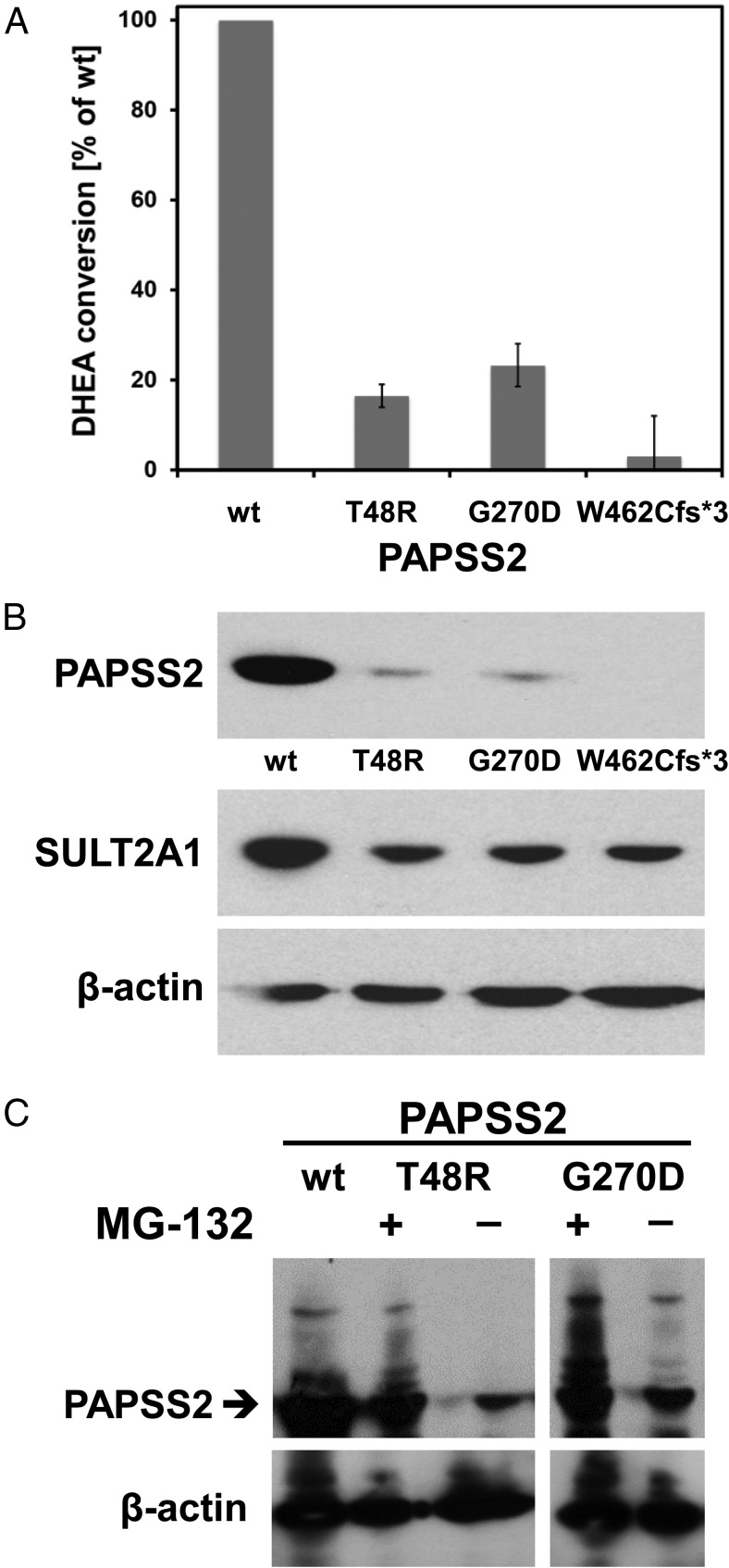
Functional in vitro assessment of the mutant PAPSS2 proteins. A, Residual enzyme activity expressed as percentage of wild-type (WT) activity, defined as 100%, based on measurements of the conversion of DHEA to DHEAS in HEK293 cells cotransfected with SULT2A1 and WT or mutant PAPSS2. Error bars represent the mean ± SEM of three independent triplicate experiments. B, Representative Western blot demonstrating equal loading and equal SULT2A1 protein expression, but significantly lower expression of mutant PAPSS2 proteins in HEK293 cells cotransfected with SULT2A1 and WT or mutant PAPSS2. C, Treatment with the proteasome inhibitor MG-132 enhances protein expression of the PAPSS2 mutants p.T48R and p.G270D, confirming increased ubiquitination of the mutant proteins.

In vitro analysis of DHEA sulfation capacity after coexpression of wild-type and mutant PAPSS2 with SULT2A1 showed a significant reduction for p.G270D, similar to the previously described p.T48R mutant (4), retaining some residual catalytic activity (Figure 2A). By contrast, coexpression of SULT2A1 with the p.W462Cfs*3 mutant almost completely abolished DHEA sulfation (Figure 2A). Western blot analysis confirmed equal SULT2A1 expression; however, the missense mutants p.T48R and p.G270D consistently showed reduced protein expression (Figure 2B), whereas mRNA expression levels determined by real-time PCR were similar (data not shown). Therefore, we reasoned that both mutations might result in destabilization of the resulting mutant PAPSS2 proteins, leading to accelerated degradation by the ubiquitin-proteasome system. To test this, we investigated whether mutant protein expression could be enhanced by the proteasome inhibitor MG-132. This was indeed the case (Figure 2C), confirming enhanced ubiquitination as an explanation for the observed reduced expression of the mutant proteins.

### In vivo analysis of DHEA sulfation by the DHEA challenge test

**Figure 3.**
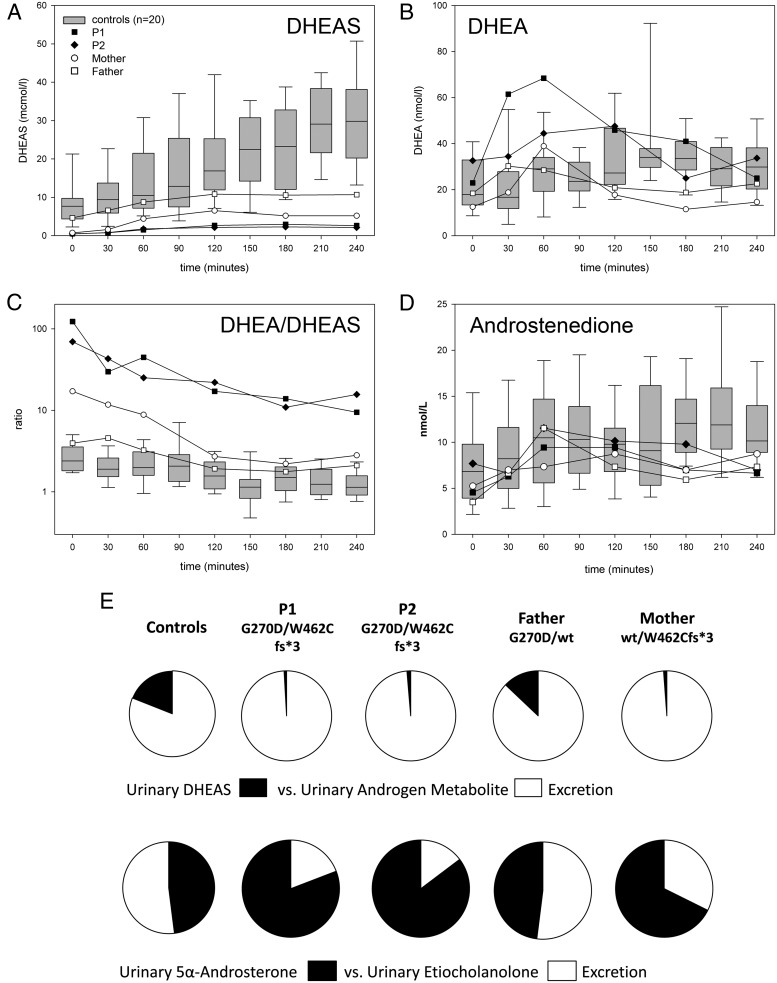
DHEA sulfation and androgen synthesis after an oral challenge with 100 mg DHEA. A–C, Serum concentrations of DHEAS (A), DHEA (B), and the ratio of serum DHEA/DHEAS (C) in the two brothers with compound PAPSS2 mutations (closed symbols) and their heterozygous parents (mother, empty circle; father, empty square) in comparison to healthy female controls (n = 20). D, Serum androstenedione after DHEA in patients, parents and healthy controls. E, Percentage of 24-hour urinary DHEAS excretion in relation to active androgen metabolite excretion after oral DHEA administration and the percentage of 5α-reduced androsterone to 5β-reduced etiocholanolone excretion, demonstrating reduced DHEAS generation and enhanced production of 5α-reduced androgens in the patients and their mother. The excretion pattern in the father resembled that observed in healthy controls (n = 8).

The two brothers carrying compound heterozygous PAPSS2 mutations and their heterozygous parents underwent in vivo assessment to scope their capacity for DHEA sulfation and androgen synthesis, utilizing an oral challenge with 100 mg DHEA with frequent serum sampling and 24-hour urine collection before and after DHEA administration (Figure 3).

At baseline, serum DHEAS was decreased in the two patients and their mother, who carries the major loss-of-function mutation p.W462Cfs*3, whereas the father (heterozygous for p.G270D) had normal DHEAS levels. Serum DHEA was in the higher normal range, and T were within the normal range for age and sex. Urinary steroid metabolite excretion analysis revealed a significantly enhanced baseline 5α-reductase activity in both patients, as represented by the ratio of 5α-reduced over 5β-reduced androgen metabolites, androsterone/etiocholanolone (An/Et) (Table 1). Similarly, their baseline excretion of 5α-reduced androgens was increased, whereas their urinary DHEAS excretion was very low. Androgen excretion in the parents was normal, except for a very low DHEAS excretion and a high 5α-reductase activity in the mother (Table 1).

**Table 1.** Urinary Steroid Metabolite Excretion at Baseline and After Oral Administration of 100 mg DHEA in the Two Brothers Affected by PAPSS2 Deficiency, in Their Heterozygous Parents, and in Healthy Controls

Urinary Steroid Metabolite	P1, G270D/W462Cfs*3	P2, G270D/W462Cfs*3	Father, G270D/WT	Mother, WT/W462Cfs*3	Postpubertal Boys, n = 10	Adult Men, n = 21	Adult Women, n = 30	Adult Women, n = 8
Age, y	17.6	15.1	48	43	14–19	25–48	25–49	23–39
24-h Urine excretion (μg/24 h) at baseline								
Androsterone	5751	4325	4450	1249	2491 (986–4928)	2286 (1332–7672)	1163 (324–2819)	893 (150–1746)
Etiocholanolone	1363	746	4819	597	1663 (313–2393)	1958 (613–5981)	1199 (507–3205)	893 (94–1990)
DHEAS	45	21	602	63	94 (31–1170)	1550 (60–6598)	280 (57–4697)	679 (49–4077)
An/Et ratio	4.22	5.80	0.92	2.09	1.71 (0.95–3.15)	1.30 (0.67–3.04)	1.03 (0.35–2.25)	1.11 (0.73–1.60)
24-h Urine excretion (μg/24 h) after oral ingestion of 100 mg DHEA								
Androsterone	35 755	35 603	13 787	12 888				4556 (778–9756)
Etiocholanolone	12 472	5982	14 650	6579				5728 (2516–12 647)
DHEAS	415	456	3444	301				6064 (463–27 835)
An/Et ratio	2.87	5.95	0.94	1.96				0.77 (0.27–1.11)

Abbreviation: WT, wild-type. Data for healthy controls are expressed as median (range).

After administration of DHEA, both patients and their mother had a subnormal rate of DHEAS generation, whereas serum DHEAS in the father increased to the lower reference range (Figure 3A). Serum DHEA peaked in both patients well above the reference range, whereas the parents showed increases comparable to controls (Figure 3B). Serum DHEA/DHEAS ratios were highly increased throughout the challenge test in both affected boys, whereas their father showed values similar to healthy controls, and the mother's results ranged in between (Figure 3C). Serum after DHEA did not differ from levels in healthy controls (Figure 3D). Serum DHT increased after oral DHEA in both boys, which was not observed in their parents. Area under the curve analysis showed reduced values for DHEAS in both patients and their mother (Table 2).

**Table 2.** Area Under the Curve of Four Sampling Hours (AUC_0–4 h_) for Serum DHEAS, DHEA, Androstenedione, T, and DHT After Oral Administration of 100 mg DHEA at Time 0 Min in the Two Brothers With PAPSS2 Deficiency, Their Heterozygous Parents, and Healthy Female Controls

Steroid AUC_0–4__h_	P1, G270D/W462Cfs*3	P2, G270D/W462Cfs*3	Father, G270D/WT	Mother, WT/W462Cfs*3	Healthy Female Controls, n = 20
Age, y	17.6	15.1	48	43	21–45
DHEAS, μmol/L*h	517	442	2268	1116	4356 (2426–9145)
DHEA, nmol/L*h	10 646	8885	5516	4688	6667 (3755–14 604)
Androstenedione, nmol/L*h	2240	1867	1825	1789	1794 (1031–3120)
T, nmol/L*h	6320	3639	7343	464	341 (72–850)
DHT, nmol/L*h	1019	983	688	^a^	^a^

Abbreviation: WT, wild-type.

a Serum DHT concentrations measured with liquid chromatography/tandem mass spectrometry were below the limit of detection in female controls. DHT levels in the heterozygous mother were detected, but below the limit of quantitation; therefore, no numbers are provided here.

Urine steroid analysis after administration of DHEA revealed significantly reduced generation of DHEAS and enhanced output of androsterone, the major metabolite of DHT, in both patients and their mother. By contrast, the father showed excretion patterns comparable to controls (Figure 3E). Androsterone excretion in the two boys was 2.6-fold higher than in their father, whereas etiocholanolone excretion was normal (Table 1); urinary excretion of 5α-17-hydroxypregnanolone, indicative of the alternative androgen pathway (22), did not increase after DHEA administration (data not shown). After DHEA ingestion, the mother showed reduced DHEAS generation and increased androsterone generation compared to healthy controls (Table 1). The An/Et ratio was significantly raised in both patients and their mother, indicative of increased 5α-reductase activity.

## Discussion

Here we carried out an integrated in vitro and in vivo analysis in a family with two brothers affected by PAPSS2 deficiency due to compound heterozygosity for two novel PAPSS2 mutations, p.W462Cfs*3s and p.G270D; their parents and sister were heterozygous carriers.

In silico analysis predicted an obvious deleterious impact of the frameshift p.W462Cfs*3 mutation due to truncation of the PAPSS2 ATP sulfurylase domain. This was confirmed in our in vitro cell-based assay, whereas the p.G270D missense mutant exhibited some residual activity. However, Western blotting demonstrated reduced protein expression for p.G270D and the previously characterized missense mutation p.T48R (4). This suggests that the mutant proteins are able to maintain some function but are subject to accelerated degradation due to reduced protein stability. We confirmed this assumption by demonstrating that inhibition of ubiquitination stabilized the mutant proteins in vitro; thus, enhanced ubiquitination represents a mechanism likely to contribute to the disruption of enzymatic activity.

Interestingly, the two affected boys had primarily presented with a severe bone phenotype, SEMD, and no apparent evidence of androgen excess. This phenotypic presentation is very similar to the first reported family with PAPSS2 deficiency, the consanguineous Pakistani family with 16 individuals affected by overt SEMD (5, 6). The bone phenotype is thought to be due to impaired proteoglycan sulfation disrupting extracellular matrix formation. The overt SEMD phenotype in the two current patients contrasts with the phenotype in the young girl we previously described (4), who presented with clinical and biochemical signs of androgen excess, manifesting as premature adrenarche and PCOS, but very mild bone abnormalities only visible on x-ray. However, all three patients had low circulating DHEAS, suggesting that in vivo DHEA sulfotransferase activity was disrupted, which should result in enhanced androgen generation from DHEA.

To examine the genotype-phenotype variation in more detail, we summarized all reported cases with PAPSS2 deficiency (Table 3). Three recent publications (8–10) have added 24 additional individuals, yielding a total of 43 patients with clinical and radiological phenotype description, whereas functional in vitro analysis of mutant protein activity was only provided by this study and our previous study (4). Table 3 illustrates that in PAPSS2 deficiency, four different bone phenotype variants are observed: 1) overt SEMD with both vertebrae and long bones affected (n = 23); 2) overt brachyolmia with dysplastic changes confined to the spine (n = 15); 3) overt brachyolmia with dysplastic changes confined to the spine with additional minimal epimetaphyseal changes only visible on x-ray (n = 4); and 4) subclinical brachyolmia with radiological changes only, as observed in the first patient we described (4).

**Table 3.** Genotype-Phenotype Spectrum in the 43 Patients With PAPSS2 Deficiency Reported to Date

First Author, Year (Ref.)	Mutant Allele 1	Mutant Allele 2	Sex	Country of Origin	Clinical Signs of Androgen Excess	Serum Androgens (Routine Biochemistry)	Bone Phenotype	Bone Age
Noordam, 2009 (4)	p.T48R	p.R329*	F	Turkey	PA, AC, HI, SA^a^	Low DHEAS; high A, T, DHT	Subclinical BO	Advanced
Iida, 2013 (10)	p.L76Q	p.R129Lfs*25	F	Turkey	AC, HI, CM	“Normal” (no details)	Overt BO	Normal
Iida, 2013 (10)	p.V540D	p.V540D	F	Turkey	AC, HI	“Normal” (no details)	Overt BO	Advanced
Iida, 2013 (10)	p.V364Rfs*18	p.V364Rfs*18	F	Turkey	No	NR	Overt BO	Normal
Iida, 2013 (10)	p.R129Lfs*25	p.R129Lfs*25	2 F	Turkey	No	NR	Overt BO	Advanced
Iida, 2013 (10)	p.R129Lfs*25	p.R129Lfs*25	F	Turkey	No	NR	Overt BO	Normal
Iida, 2013 (10)	p.Q211Cfs*11	p.Q211Cfs*11	F	Turkey	No	NR	Overt BO	Normal
Iida, 2013 (10)	c.27 + 3A>C	c.27 + 3A>C	F	Turkey	No	NR	Overt BO	Normal
Iida, 2013 (10)	p.F125Sfs*24	p.F125Sfs*24	F	Syria	PP	High DHEA	Overt BO	Advanced
Miyake, 2012 (8)	p.A113Gfs*18	p.A113Gfs*18	F	Turkey	No	NR	Overt BO	NR
Miyake, 2012 (8)	p.A113Gfs*18	p.A113Gfs*18	F	Turkey	No	Low DHEAS	Overt BO, MEMC	Advanced
Miyake, 2012 (8)	p.V206Sfs*9	p.R437Gfs*19	F	Japan	No	NR	Overt BO, MEMC	Advanced
Miyake, 2012 (8)	c.381 + 2delT	c.381 + 2delT	F	Japan	No	NR	Overt BO, MEMC	Advanced
Tüysüz, 2013 (9)	p.R329*	p.R329*	F	Turkey	HI, OM	Low DHEAS, normal DHEA, A, T	Overt SEMD	NR
Tüysüz, 2013 (9)	p.R329*	p.R329*	F	Turkey	No	Low DHEAS, normal DHEA, A, T	Overt SEMD	NR
Ahmad, 1998 (6)	p.S480*	p.S480*	11 M, 5 F	Pakistan	NR	NR	Overt SEMD	NR
Iida, 2013 (10)	p.L76Q	p.R129Lfs*25	M	Turkey	No	NR	Overt BO	Normal
Iida, 2013 (10)	p.C43Y	p.C43Y	M	Turkey	PA	NR	Overt BO	Advanced
Iida, 2013 (10)	p.V364Rfs*18	p.V364Rfs*18	M	Turkey	No	NR	Overt BO	Advanced
Iida, 2013 (10)	p.A113Gfs*18	p.A113Gfs*18	2 M	Turkey	No	NR	Overt BO	NR
Miyake, 2012 (8)	p.A113Gfs*18	p.A113Gfs*18	M	Turkey	No	NR	Overt BO	Delayed
Miyake, 2012 (8)	p.K161Rfs*6	p.I221Sfs*40	M	Korea	No	NR	Overt BO, MEMC	Advanced
Tüysüz, 2013 (9)	p.R329*	p.R329*	3 M	Turkey	No	Low DHEAS, normal DHEA, A, T	Overt SEMD	NR
Present study	p.G270D	p.W462Cfs*3	2 M	Netherlands	No^b^	Low DHEAS, normal DHEA, A, T	Overt SEMD	Delayed

Abbreviations: F, female; M, male; NR, not reported; PA, premature adrenarche; AC, acne; HI, hirsutism; SA, secondary amenorrhea; CM, clitoromegaly; PP, precocious puberty; OM, oligomenorrhea; A, androstenedione; BO, brachyolmia (short trunk-short stature due to platyspondyly); MEMC, minimal epimetaphyseal changes; SEMD, spondyloepimetaphyseal dysplasia (brachyolmia plus additional clinically overt epimetaphyeseal changes). Overt indicates clinical and radiological changes, and subclinical indicates radiological changes only. Mutation nomenclature is according to Human Genome Variation Society convention and employs *PAPSS2* reference sequence NM_001015880.1.

a Mother reported PCOS with oligomenorrhea and anovulation.

b Mother reported chronic anovulation requiring ovulation induction with clomiphene for conception; older sister had primary amenorrhea.

Only 16 of the 43 patients reported to date were assessed for androgen excess; clinical signs including premature adrenarche and PCOS were reported for six of them (Table 3). Serum DHEAS was measured in eight patients and invariably found to be low. Circulating androgens were measured in 10 individuals and increased in two female patients, whereas eight patients had normal androgens (four males, four females) (Table 3). Although circulating androgens were normal in the two affected male patients in this study, urinary steroid metabolite analysis revealed increased 5α-reduced androgens in both of them, indicative of enhanced generation of active androgens in peripheral tissues from DHEA. We confirmed this employing oral administration of the androgen precursor DHEA to assess DHEA sulfation and androgen synthesis capacity, which demonstrated that in the two patients, DHEA was converted to active androgens at a much higher rate than in their father or in healthy controls. The biological consequences of this androgen excess will need to be monitored prospectively, and it is interesting to note that neither boy had signs of premature adrenarche. Our control group consisted of adult women; however, we demonstrated previously that the conversion pattern toward DHEA, DHEAS, and after oral DHEA is identical in men and women (18, 23). In healthy men, DHEA administration does not elicit increases in T or DHT (23); therefore, it is interesting to note that both patients showed a clear increase in circulating DHT after oral DHEA. Using an oral DHEA challenge as a dynamic test, we provided the first direct functional in vivo evidence that impaired DHEA sulfation results in androgen excess.

The mother of the two patients studied here reported clinical features consistent with a PCOS phenotype, specifically chronic anovulation requiring ovulation induction. Androgen analysis showed evidence of enhanced 5α-reductase activity at baseline and increased 5α-reduced androgen production after the oral DHEA challenge, with low DHEAS at baseline and subnormal DHEAS generation after DHEA. The mother of our first patient (4) had actually reported a history of PCOS with oligomenorrhea and anovulation, but at the time had not provided serum or urine for more detailed androgen analysis. Coincidentally, both mothers were heterozygous carriers of a major loss-of-function *PAPSS2* mutation, and thus it appears reasonable to assume that heterozygosity for a mutant allele does not only result in a subclinical phenotype with low DHEAS but can also impact clinically with features resembling PCOS. Two recent studies looked at the association of common genetic variants (minor allele frequency [MAF] > 5%) in *SULT2A1* and *PAPSS2* with androgen status (24, 25); future studies with a more detailed phenotyping and genotyping approach will need to investigate impaired DHEA sulfation as a predisposing factor to PCOS by looking for low frequency (MAF < 5%) and rare variants (MAF < 0.5%).

In summary, we have presented conclusive in vivo evidence for androgen excess as a consequence of impaired DHEA sulfation in PAPSS2 deficiency. Further studies are needed to determine the effect on drug metabolism in affected patients because sulfation is a key process in the inactivation of drugs and xenobiotics, which may explain the multiple drug intolerances reported by the mother. Studies in PCOS cohorts will need to look more closely at the pattern of androgen excess as a differentiating factor, with mounting evidence that androgen status is closely linked to metabolic risk in PCOS patients (20).
